# The effects of taxanes, vorinostat and doxorubicin on growth and proliferation of *Echinococcus multilocularis* metacestodes assessed with magnetic resonance imaging and simultaneous positron emission tomography

**DOI:** 10.18632/oncotarget.24142

**Published:** 2018-01-10

**Authors:** Xiangsheng Huang, Stefan Wiehr, Anna-Maria Wild, Patrick Voßberg, Wolfgang Hoffmann, Beate Grüner, Carsten Köhler, Peter T. Soboslay

**Affiliations:** ^1^ Institute for Tropical Medicine, Eberhard Karls University, Tübingen, Germany; ^2^ Werner Siemens Imaging Center, Department of Preclinical Imaging and Radiopharmacy, Eberhard Karls University, Tübingen, Germany; ^3^ Section of Clinical Immunology and Infectiology, University Clinics Ulm, Ulm, Germany

**Keywords:** echinococcus multilocularis, metacestode, taxanes, paclitaxel, docetaxel, histone deacetylase inhibitor, vorinostat, doxorubicin, drug exposure, positron emission tomography, magnetic resonance imaging

## Abstract

Cytostatic drugs used in cancer therapy were evaluated for their capacity to inhibit *Echinococcus multilocularis* metacestode growth and proliferation. Metacestode tissues were exposed *in vitro* to docetaxel, doxorubicin, navelbine, paclitaxel, and vorinostat for 1 week, then incubated in drug-free culture, and thereafter metacestodes were injected into the peritoneum of *Meriones unguiculatus*. Magnetic resonance imaging (MRI) and simultaneous positron emission tomography (PET) were applied to monitor *in vivo* growth of drug-exposed *E. multilocularis* in *Meriones*. At 3 month p.i., docetaxel (at 10 μM, 5 μM and 2 μM) inhibited *in vivo* growth and proliferation of *E. multilocularis*, and at 5 months p.i., only in the 2 μM docetaxel exposure group 0.3 cm**^3^** of parasite tissue was found. With paclitaxel and navelbine the *in vivo* growth of metacestodes was suppressed until 3 months p.i., thereafter, parasite tissues enlarged up to 3 cm**^3^** in both groups. *E. multilocularis* tissues of more than 10 g developed in *Meriones* injected with metacestodes which were previously exposed *in vitro* to doxorubicin, navelbine, paclitaxel or vorinostat. In *Meriones* infected with metacestodes previously exposed to docetaxel, the *in vivo* grown parasite tissues weighted 0.2 g. *In vitro* cultured *E. multilocularis* metacestodes exposed to docetaxel did not produce vesicles until 7 weeks post drug exposure, while metacestodes exposed to doxorubicin, navelbine and vorinostat proliferated continuously. In summary, docetaxel, and less efficaciously paclitaxel, inhibited *in vivo* and *in vitro* parasite growth and proliferation, and these observations suggest further experimental studies with selected drug combinations which may translate into new treatment options against alveolar echinococcosis.

## INTRODUCTION

Alveolar echinococcosis (AE), a life-threatening zoonosis for humans, is caused by the proliferative growth of the larval metacestode of *Echinococcus multilocularis* (Em) within tissues and organs, mostly the liver [1]. Surgical removal of the infested organs or tissues and long-lasting benzimidazole (BMZ) therapy will improve the survival rate of patients, however, the chemotherapeutic options remain limited and new treatments of AE are needed. Long-lasting BMZ treatment is parasitostatic and not parasitocidal, and as such, despite surgical resection of parasite tissues, undetected and residual larval metacestodes may restart growth with progression of disease as soon as chemotherapy is stopped.

*E. multilocularis* can be maintained in an experimental life cycle by intra-peritoneal inoculation of larval metacestodes into permissive hosts such as *Meriones unguiculatus* (gerbils). The metacestode larvae will progressively grow in gerbils and parasite tissues can be collected and used for research and diagnostic purposes. To evaluate the efficacy of chemotherapy, *E. multilocularis* infected gerbils can be treated with parasiticides or cytostatic drugs [2–4]. *In vitro* cultured metacestodes can selectively be exposed to anti-helminthic drugs or new compounds to evaluate their parasitocidal or parasitostatic efficacy [5–9], or else, after intra-peritoneal transfer of these drug-exposed metacestodes into permissive recipients, e.g. gerbils, the viability and proliferative capacity of the parasite tissues can be evaluated *in vivo* [10–12]. Cytostatic drugs used in cancer therapy were applied to determine their potential to inhibit *E. multilocularis* metacestode growth and proliferation [2–4, 7, 9, 11, 12]. The selection of cytostatic drugs was based on gene expression analysis of the *E. multilocularis* metacestode tissue, which disclosed that metacestodes expressed genes associated with proliferation of cancer cells and progressive tumor growth, which can be inhibited by specific anti-cancer compounds [11]. Inhibitors of tubulin genes were chosen for this study. The taxanes (docetaxel, paclitaxel) and vinorelbine (navelbine) are microtubule-stabilizing agents that function primarily by interfering with spindle microtubule dynamics causing cell cycle arrest and apoptosis [13]. Paclitaxel at clinically achievable concentrations inhibited *in vitro* the survival of larval cells, protoscoleces and metacestodes of *Echinococcus granulosus* [9], while metacestode vesicles of *E. multilocularis* when *in vitro* cultured and exposed to paclitaxel, docetaxel or vorinostat were not affected [12]. Navelbine has been tested *in vivo* against *E. multilocularis* by Hübner *et al.* 2010, and *in vitro* by Stadelmann *et al*. 2014, and the drug did not did not show parasitocidal or clear parasitostatic effects [11, 12]. Vorinostat (SAHA) is one of the most potent inhibitors of histone acetyltransferases and histone deacetylases (HDAC) and clinical trials have shown it to be effective against cutaneous T-cell lymphoma and other malignancies [14]. The anti-cancer agent doxorubicin is a membrane permeable drug which mediates DNA damage and inhibits DNA synthesis, promotes reactive oxygen species and cell senescence, it will cause cardiotoxicity and drug resistance while being of low bio-availability [15]. With doxorubicin, when bound to bio-degradable nanoparticles and applied into *E. multilocularis* infected mice, the hepatic parasite development and metacestode viability were reduced, but free doxorubicin had no anti-parasitic activity [4]. For the pre-clinical evaluations of therapeutic effects of tumor suppressors in various types of cancers, *in vivo* positron emission tomography (PET) combined with *ex vivo* histology and nuclear magnetic resonance (NMR) metabolic fingerprinting was successfully applied for therapy monitoring [16, 17]. Such *in vivo* imaging techniques have also been used for non-invasive diagnosis of invasive pulmonary aspergillosis [18]. The follow-up of patients with AE was accomplished with delayed glucose traced-assisted PET which facilitated the differentiation between active and inactive liver lesions [19]. In experimental animal models of AE magnetic resonance imaging [20] or ultrasound [21] were successfully applied to follow-up parasite growth in living animals during the treatment phase.

In this study, cytostatic drugs at present used in cancer therapy were evaluated for their capacity to inhibit *E. multilocularis* metacestode growth and proliferation. We have exposed *in vitro* parasite tissues to drug concentrations used for the therapy of cancer patients, this was to evaluate the parasitostatic or parasitocidal efficacy of these cytostatic drugs at concentrations not applicable and adapted for *in vivo* use with experimental animals. After the *in vitro* exposure, and one week of culture in drug-free medium to wash out residual drug from the parasite tissue blocks, the *E. multilocularis* metacestode tissues were injected into parasite-susceptible animals (*Meriones unguiculatus*, gerbils) and this approach evaluated whether the preceding drug-exposure would inhibit *in vivo* parasite growth or has had a parasitocidal effect. Magnetic resonance imaging (MRI) and simultaneous positron emission tomography (PET) with the 2-deoxy-2-[^18^F]-fluoro-D-glucose ([^18^F]FDG) tracer were applied [20] to monitor *in vivo* the growth of drug-exposed *E. multilocularis* metacestodes, and in parallel, drug-exposed parasite tissues were studied *in vitro* for growth and proliferative “budding” of metacestode vesicles.

## RESULTS

### Selection of cytostatic drugs for *in vitro* exposure with *E. multilocularis* metacestodes

The analysis of *E. multilocularis* cDNA hybridization to human microarrays showed strongly expressed cancer-related genes in metacestodes. The signal strength of hybridization of *E. multilocularis* cDNA to human genes was prominent for member of the RAS oncogene family (RAB2), the folate receptor (FOLR1), the eukaryotic translation elongation factor 1 alpha 1 (EEF1A1), tubulins (TUBA1A, TUBA1C, TUBB3), aquaporin, calreticulin, and synuclein alpha (Table 1 PartA, Supplementary Table 1 PartB). These microarray results suggested similarities between *E. multilocularis* metacestode proliferation and cancer progression and tissue metastases, and thus, the selection of drugs for the evaluation of their capacity to inhibit *E. multilocularis* growth and proliferation, was on FDA-approved compounds that disrupt normal function of microtubules and interfere with the cell division or replication. The taxanes docetaxel, paclitaxel and navelbine and also the histone deacetylase inhibitor SAHA (vorinostat) as well as doxorubicin were investigated in this study. *E. multilocularis* metacestodes were exposed *in vitro* to cytostatic drugs, then those drug-exposed parasite tissues were injected into *Meriones unguiculatus* (gerbils), and parasite growth and proliferation studied by MRI and PET.

**Table 1A:** The signal strength of hybridization of Echinococcus multilocularis cDNA to human microarray chip

Genbank Accession No.	Signal Strength Sample 1	Signal Strength Sample 2	Mean Signal Strength (S1 + S2)	Gene Title
AA535244	1.050	837,5	944	RAB2, member RAS oncogene family
AL515273	499,6	433,1	466	eukaryotic translation elongation factor 1 alpha 1
AK098740	440,4	468,6	455	hypothetical protein LOC202051
BE221212	631,5	140,3	386	collagen, type I, alpha 1
L36675	414,8	305,7	360	synuclein, alpha (non A4 component of amyloid precursor)
AJ006206	366,8	308,6	338	B1 for mucin /// similar to MUC-B1
AL581768	399,7	244,6	322	tubulin, alpha, ubiquitous
AF000381	376	229,3	303	folate receptor 1 (adult)
AW015506	284,9	298,9	292	aquaporin 2 (collecting duct)
NM_001402	294,3	237,2	266	eukaryotic translation elongation factor 1 alpha 1
BE964125	322,6	207,2	265	similar to eukaryotic translation elongation factor 1 alpha 1; eukaryotic translation elongation factor 1 alpha 1-like 14; CTCL tumor antigen; translation elongation factor 1 alpha 1-like 14; prostate tumor-inducing protein 1; EF1a-like protein;
AL137719	257,8	248,2	253	olfactory receptor, family 7, subfamily E, member 104 pseudogene
BE786672	299,2	203,3	251	eukaryotic translation elongation factor 1 alpha 1
AK098354	292,7	208,7	251	BS 3076
AI378706	214,6	204,5	210	Calreticulin
AW001777	235,1	176	206	hypothetical LOC400843
U15197	283	127,6	205	ABO blood group (transferase A, alpha 1-3-N-acetylgalactosaminyltransferase; transferase B, alpha 1-3-galactosyltransferase)
AW271225	220,4	163,1	192	oxysterol binding protein-like 5
BI912454	233,1	119,5	176	hypothetical locus LOC338799
AK096064	210	141,1	176	---
NM_024732	194,1	154,1	174	hypothetical protein FLJ14351
NM_152909	208,1	129,4	169	zinc finger protein 548
AW612342	196,9	127,3	162	Rho-associated, coiled-coil containing protein kinase 1
AK093104	179,3	123	151	hypothetical protein FLJ35785
AL133228	198,3	100,8	150	thymosin, beta 4, X-linked /// thymosin-like 3
AI820801	203,9	82,7	143	Transcribed locus
Z22814	155,6	124,7	140	atrophin 1
NM_153606	182,4	86,8	135	family with sequence similarity 71, member A
BF223582	180,9	87,9	134	---
AV710357	196	70,9	133	---
AA046650	172,7	89	131	TRIO and F-actin binding protein
BC005946	185,9	75,2	131	tubulin alpha 6 /// tubulin alpha 6
NM_001403	81,4	172	127	eukaryotic translation elongation factor 1 alpha 1
NM_014030	127,9	122	125	G protein-coupled receptor kinase interactor 1
BC004949	141,2	107,3	124	tubulin alpha 6
AI869532	113,6	131,1	122	Nuclear factor related to kappaB binding protein
W07773	109,2	129,4	119	chromosome 19 open reading frame 22
BU928170	139,2	98,4	119	Similar to F4N2.10
NM_001030	151,2	85,1	118	ribosomal protein S27 (metallopanstimulin 1)
BC013641	124,5	107,3	116	Homo sapiens, clone IMAGE:4151631, mRNA
Y15916	110,9	112,5	112	collagen, type I, alpha 1
AJ251708	177,6	45,1	111	putative microtubule-binding protein
U58856	108,5	114	111	mannose receptor, C type 2
AW015517	95,3	126,6	111	Follistatin-like 3 (secreted glycoprotein)
AL565749	125,1	94,71	110	tubulin, beta 3
AB009010	147,3	72,4	110	ubiquitin C
AJ296370	168,8	50	109	---
BC015443	99,21	119,1	109	Pseudogene similar to LOC112869 gene
BE300252	140,7	77,2	109	tubulin, alpha, ubiquitous
AL031186	126,6	89,59	108	EMI domain containing 1
BF246436	133,1	78,3	106	eukaryotic translation initiation factor 1
AF343666	112,8	97,7	105	Translocation associated fusion protein IRTA1/IGA1 (IRTA1/IGHA1) /// Translocation associated fusion protein IRTA1/IGA1 (IRTA1/IGHA1)
BC004952	129,4	75,3	102	polycomb group ring finger 1
AW974499	99,1	103,4	101	Rho GTPase activating protein 30
AI885873	122,4	78	100	transportin 2 (importin 3, karyopherin beta 2b)
BE552347	108,6	91,8	100	Kv channel interacting protein 2
BE813017	120,3	79,3	100	---

### *In vivo* growth of drug-exposed *E. multilocularis* in Meriones unguiculatus

**Figure 1:**
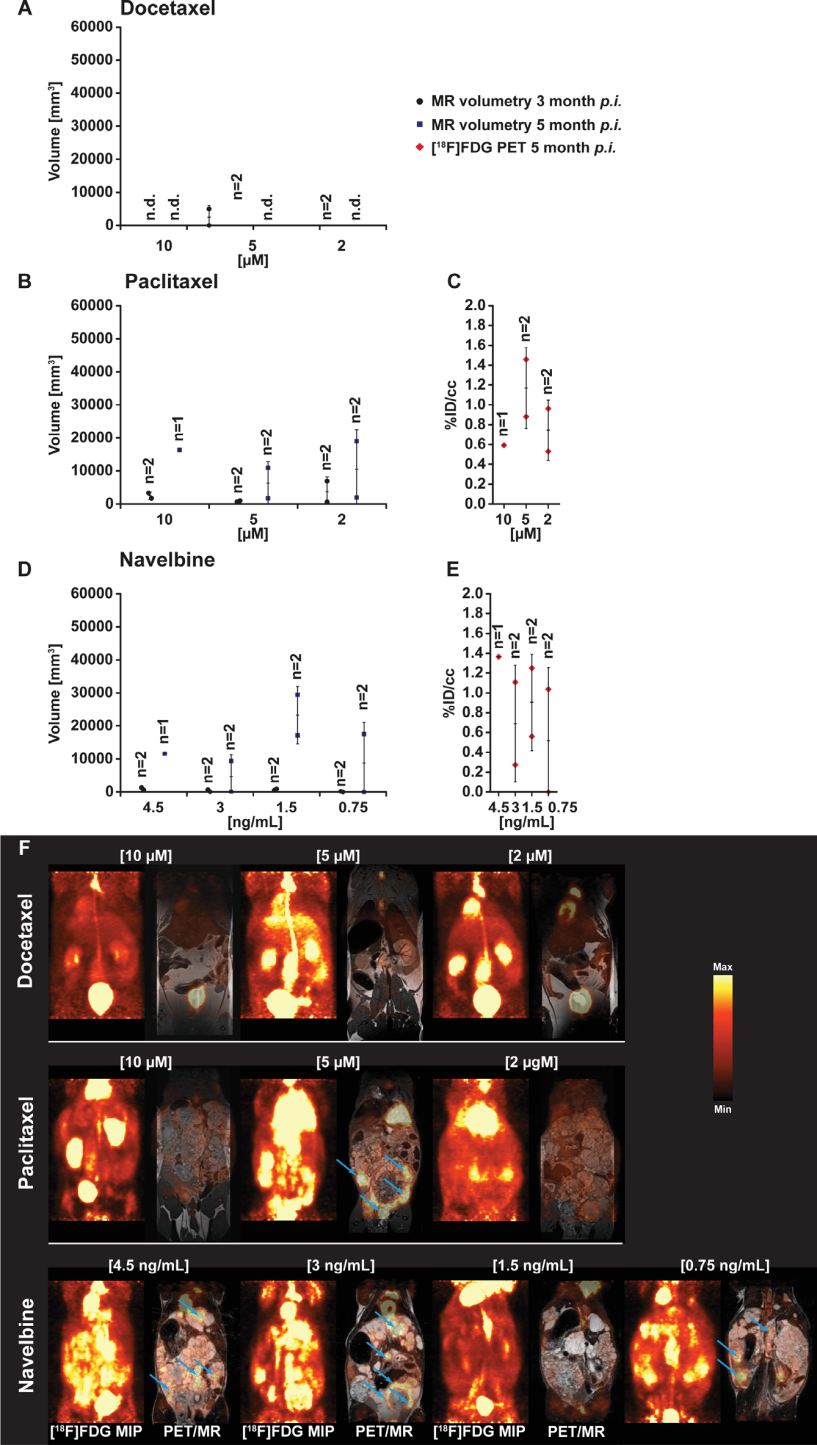
PET- and MR-imaging, and quantification of parasite growth, in docetaxel, paclitaxel and vorinostat (SAHA) exposed *E.multilocularis* metacestodes *In vivo* quantification of parasite growth was performed at two time points in all tested animals. The *in vivo* grown volumes of docetaxel (**A**), paclitaxel (**B**) and navelbine (**D**) exposed *E. multilocularis* metacestodes is shown. At the second measurement time point (**F**) and simultaneously to the MR acquisition all animals were PET imaged with [^18^F]FDG. Quantification of the [^18^F]FDG uptake in parasite tissue is presented as percentage of the injected dose per cubic centimeter (%ID/cc) and error bars represent one SD. Results are shown for docetaxel (**C**), paclitaxel and navelbine (**E**) exposure. Coronal [^18^F]FDG maximum intensity projections (MIP) and fused PET/MR images from *E. multilocularis* metacestode infected gerbils are shown in the Figure 1F. Arrows indicate the positions of the [^18^F]FDG uptake in the metacestode tissue. (n.d., non detected).

*In vivo* volumetric MRI measurements were performed at two time points after inoculation of drug-exposed *E. multilocularis* metacestode tissues into *Meriones*. Analysis of the MR images showed low or no parasite growth in infected *Meriones* if metacestodes were exposed to docetaxel *in vitro*, independent of the applied concentration (Figure 1A and 1F). At 3 month post transfer of drug-exposed metacestodes in *Meriones*, the MRI measurement detected 0 mm^3^ (*n* = 2), 0 and 4934 mm^3^ (*n* = 2) and 0 and 6 mm^3^ (*n* = 2) of parasite tissues in the docetaxel 10 μM, 5 μM and 2 μM exposure groups, respectively. At 5 months p.i., the MRI measurement did not detect any metacestodes in the docetaxel 10 μM 5 μM and 2 μM exposure groups.

The *in vitro* exposure of metacestode tissues to paclitaxel did not prevent their growth after transfer into the peritoneum of *Meriones* (Figure 1B and 1F). At 3 months p.i., metacestodes which were *in vitro* exposed to paclitaxel at concentrations of 10 μM, 5 μM and 2 μM developed in *Meriones* into parasite tissues with volumes 1694 and 3316 mm^3^ (*n* = 2); 973 and 619 mm^3^ (*n* = 2) and 6932 and 577 mm^3^ (*n* = 2), respectively. At 5 months p.i. the MRI measurement determined 16370 mm^3^ (*n* = 1), 1692 and 10931 mm^3^ (*n* = 2) and 1933 and 18977 mm^3^ (*n* = 2) of parasite tissues in the paclitaxel 10 μM, 5 μgM and 2 μM exposure groups, respectively. Following navelbine exposure, small metacestode tissue volumes were detected at 2 months post transfer in *Meriones*, but parasite volumes enlarged at 5 months post transfer (Figure 1D and 1F).

When exposed to navelbine at concentrations of 4.5 ng/ml, 3 ng/ml 1.5 ng/ml and 0.75 ng/ml, the tissue volumes detected by MRI at 2 months p.i. were 570 and 1290 mm^3^ (*n* = 2), 51 and 636 mm^3^ (*n* = 2), 890 and 468 mm^3^ (*n* = 2) and 0 and 177 mm^3^ (*n* = 2), respectively. At 5 months p.i., parasite tissue volumes of 11565 mm^3^ (*n* = 1), 78 and 9337 mm^3^ (*n* = 2), 17151 and 29422 mm^3^ (*n* = 2) and 0 and 17496 mm^3^ (*n* = 2) have grown in *Meriones* when the navelbine exposure concentrations were 4.5 ng/ml, 3 ng/ml 1.5 ng/ml and 0.75 ng/ml, respectively.

**Figure 2:**
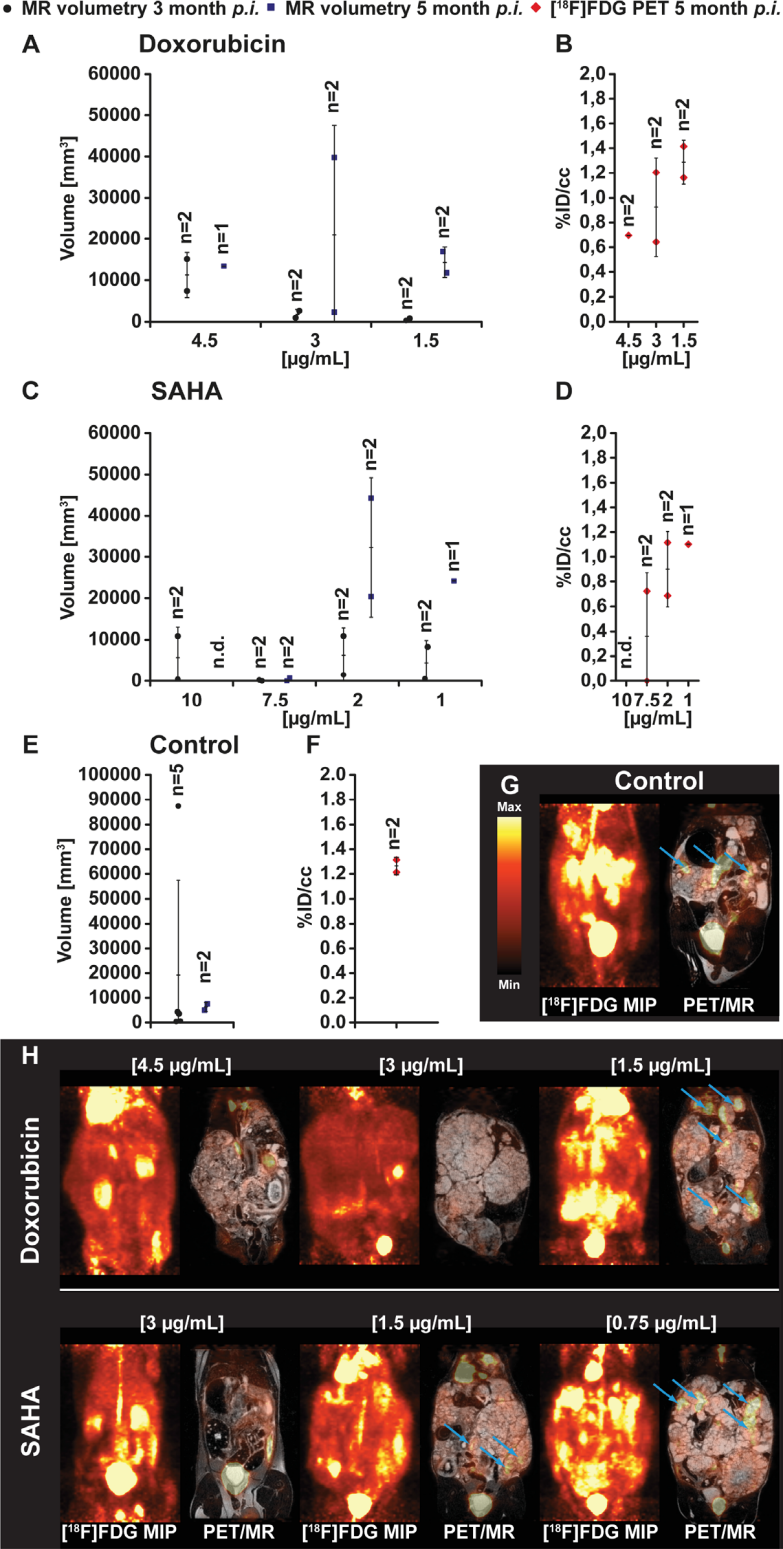
PET- and MR-imaging, and quantification of parasite volumes, in doxorubicin, vorinostat (SAHA) and DMSO (control) exposed *E. multilocularis* metacestodes *In vivo* quantification of parasite growth was performed at two time points in all tested animals. The parasite tissue volumes of doxorubicin (**A**), vorinostat (SAHA; **C**) and DMSO control (**E**) exposed *E. multilocularis* metacestodes is shown. At the second measurement time point and simultaneously to the MR acquisition all animals were PET imaged with [^18^F]FDG. Quantification of the [^18^F]FDG uptake in parasite tissue is presented as percentage of the injected dose per cubic centimeter (%ID/cc) and error bars represent one SD. Results are shown for doxorubicin (**B**), vorinostat (SAHA; **D**) and DMSO control (**F**) treatment. Coronal [^18^F]FDG maximum intensity projections (MIP) and fused PET/MR images from *E. multilocularis* metacestode infected gerbils are depicted in the (**G** and **H**). Arrows indicate the positions of the [^18^F]FDG uptake in the metacestode tissue.

Animals inoculated with *E. multilocularis* metacestodes exposed to doxorubicin (Figure 2A and 2H) showed comparable parasite growth as seen in the group treated with paclitaxel. At 2 months post inoculation, metacestodes exposed *in vitro* to doxorubicin at concentrations of 4.5 μg/ml, 3 μg/ml and 1.5 μg/ml developed *in vivo* in *Meriones* parasite tissue volumes of 7385 and 15138 mm^3^ (*n* = 2), 2623 and 967 mm^3^ (*n* = 2) and 810 and 332 mm^3^ (*n* = 2), respectively. At 5 months p.i., tissue volumes of 13426 mm^3^ (*n* = 1), 39768 and 2297 mm^3^ (*n* = 2) and 11733 and 16953 mm^3^ (*n* = 2) were present in the respective doxorubicin exposure groups.

*In vivo* growth of *E. multilocularis* metacestode was observed following *in vitro* exposure with vorinostat (SAHA) (Figure 2C and 2H). At 3 months post inoculation in *Meriones,* parasite tissues with volumes of 10879 and 412 mm^3^ (*n* = 2), 0 and 220 mm^3^ (*n* = 1), 10887 and 1470 mm^3^ (*n* = 2) and 8204 and 521 mm^3^ (*n* = 2) have developed from vorinostat (SAHA) 10 μg/ml, 7.5 μg/ml, 5 μg/ml, 2.5 μg/ml exposed metacestodes, respectively. At 5 months post inoculation, MRI measurements could be conducted in *Meriones* with vorinostat (SAHA) 7.5 μg/ml, 2 μg/ml and 1 μg/ml exposed metacestodes, and 755 and 0 mm^3^ (*n* = 2), 20389 and 44256 mm^3^ (*n* = 2) and 24190 mm^3^ (*n* = 1) of tissues were found, respectively.

The *in vitro* DMSO-exposed metacestode tissues which were transferred into *Meriones* were prominently enlarged *in vivo* at the first time point of measurement with 19264 ± 38127 mm^3^ (*n* = 5; at 3 months post transfer) and on the second measurement 7606 and 5049 mm^3^ of parasite tissue was detected (*n* = 2; at 5 months post transfer) (Figure 2E and 2G control). Due to strong parasite growth in the DMSO control group, 3 animals had to be euthanized according to the animal welfare guidelines which resulted in a lower mean parasite burden at the second measurement time point.

### *In vivo* positron emission tomography (PET) and magnetic resonanz imaging (MRI) of Meriones unguiculatus infected with drug-exposed E. multilocularis

For the *in vivo* evaluation of the glucose metabolism, *E. multilocularis* infected and control *Meriones* were injected with the PET tracer [^18^F]FDG for glucose consumption, and PET/MRI were simultaneously performed at 5 months post infection in one set of experiments. When *E. multilocularis* metacestodes were exposed *in vitro* to docetaxel and these metacestodes then transferred into the peritoneum of *Meriones*, the PET quantification showed no uptake of [^18^F]FDG in these animals, independent of the *in vitro* applied concentrations of docetaxel. An increased [^18^F]FDG tracer uptake of 0.6 %ID/cc (*n* = 1), 0.9 and 1.5 %ID/cc (*n* = 2) and 0.5 and 1.0 %ID/cc was seen in gerbils implanted with metacestode tissue exposed to paclitaxel at concentrations of 10 μg/ml, 5 μg/ml and 2 μg/ml, respectively (Figure 1C). 1F *Meriones* inoculated with navelbine-exposed *E. multilocularis* metacestodes, the [^18^F]FDG tracer uptake into parasite tissues was heterogeneous when compared to vorinostat and doxorubicin. Tracer uptake was 1.4 %ID/cc (*n* = 1), 0.3 and 1.1 %ID/cc (*n* = 2); 1.3 and 0.6 %ID/cc (*n* = 2) and 0 and 1.0 %ID/cc (*n* = 2) when metacestodes were exposed *in vitro* to navelbine at concentrations of 4.5 μg/ml, 3 μg/ml, 1.5 μg/ml and 0.75 μg/ml, respectively (Figure 1D).

The preceding *in vitro* exposure of metacestodes to doxorubicin led to a dose dependent uptake of [^18^F]FDG in parasite tissue in *Meriones* with 0.7 %ID/cc (*n* = 1), 0.6 and 1.2 %ID/cc (*n* = 2), and 1.4 and 1.2 %ID/cc (*n* = 2) when doxorubicin was applied at concentrations of 4.5 μg/ml, 3 μg/ml and 1.5 μg/ml, respectively (Figure 2B). Similarly, in *Meriones*, a dose dependent uptake of the [^18^F]FDG tracer in *E. multilocularis* metacestode tissue was detected when the preceding *in vitro* exposure was with vorinostat (SAHA). Animals inoculated with *E. multilocularis* metacestodes treated with 10 μg/ml vorinostat showed no uptake of [^18^F]FDG due to no parasite growth. Exposure with 7.5 μg/ml of vorinostat resulted in low [^18^F] FDG uptake with 0 and 0.7 %ID/cc (*n* = 2); when metacestodes were exposed to 5 μg/ml vorinostat then tracer uptake was 1.1 and 0.7 %ID/cc (*n* = 2) and exposure with 2 μg/ml showed a tracer uptake of 1.1 %ID/cc (Figure 2D). In *Meriones* transferred with DMSO-exposed *E. multilocularis* metacestodes (positive control) the uptake of [^18^F]FDG was at 1.3 and 1.2 %ID/cc (*n* = 2) (Figure 2F).

### The *in vivo* weights of E. multilocularis metacestodes after *in vitro* drug-exposure

**Figure 3:**
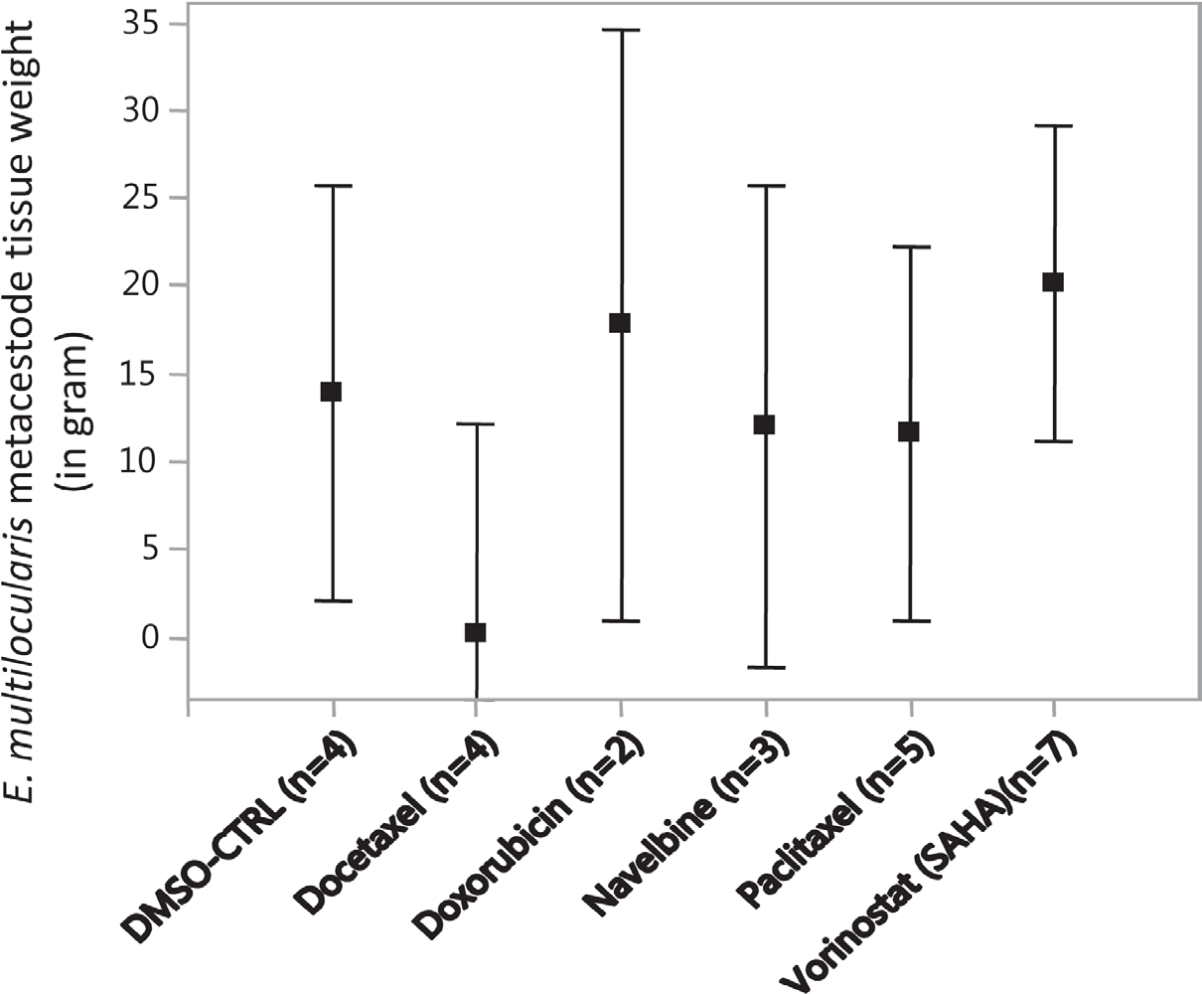
The weights of *E. multilocularis* metacestodes tissues isolated from infected *Meriones unguiculatus* Metacestodes were exposed *in vitro* to the cytostatic drugs docetaxel (10 μM, 5 μM, 2 μM), doxorubicin (4.5, 3 and 1.5 μg/ml), navelbine (4.5, 3, 1.5 and 0.75 μg/ml), paclitaxel (10 μM, 5 μM and 2 μM), vorinostat (SAHA) (10, 7.5, 2 and 1 μg/ml) and DMSO (0.1%, 0.05%, solvent control, CTRL) at the indicated concentrations for 7 days, subsequently metacestodes rested in drug-free media for another 7 days, and then the drug-exposed metacestodes were injected into the peritoneum of *M. unguiculatus*. At 4 and 5 months post infection, the grown metacestode tissues were collected from *M. unguiculatus* and weighted. The drug concentration groups at which *E. multilocularis* metacestodes tissues were exposed to the cytostatic drugs are merged. The Figure shows the treatment groups, the mean metacestode tissue weights and the 95% confidence intervals. No significant differences in weights were observable between the treatment groups.

After the final PET scan at 5 months post infection, all *Meriones* were euthanized under deep anesthesia and parasite tissues were removed and weighted. *E. multilocularis* metacestode tissue masses of more than 10 g developed in *Meriones* injected with metacestodes which were previously exposed *in vitro* to doxorubicin (mean parasite tissue weight: 17.8 g), navelbine (12.0 g), paclitaxel (11.6 g) or vorinostat (SAHA) (20.1 g), while in those animals which were injected with metacestodes previously exposed to docetaxel, the *in vivo* grown metacestodes weighted 0.2 g (Figure 3). In Figure 3 the weights of the *in vivo* grown metacestode tissues from the animal groups with selected drug concentrations were merged.

### The *in vitro* production “budding” of vesicles from E. multilocularis metacestode tissues after *in vitro* drug-exposure

**Figure 4:**
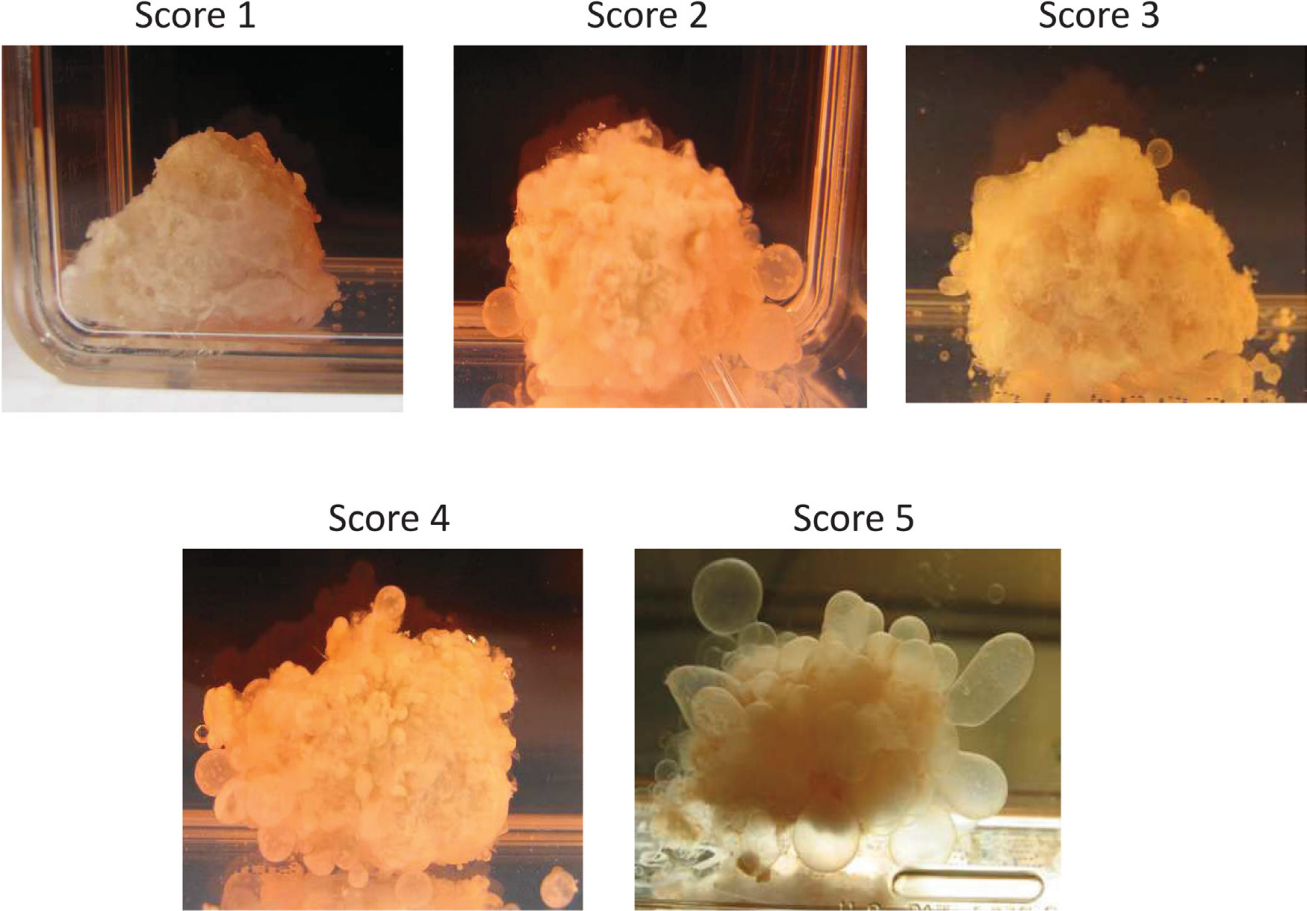
The *in vitro* “budding” of vesicles from E. multilocularis metacestode tissues after drug-exposure The “budding” of *E. multilocularis* vesicles from *in vitro* cultured metacestodes tissue previously exposed *in vitro* to cytostatic drugs was evaluated during 14 weeks post drug exposure. The number of vesicles produced in culture was scored, i.e. Score 0 = no vesicle, Score 1 = very few vesicles (1–5), Score 2 = few vesicles (6–10), Score 3 = vesicles (11–20), Score 4 = vesicles (21–30), Score 5 = vesicles (>31). The production scores 1–5 of *E. multilocularis* metacestodes tissues are shown.

**Figure 5:**
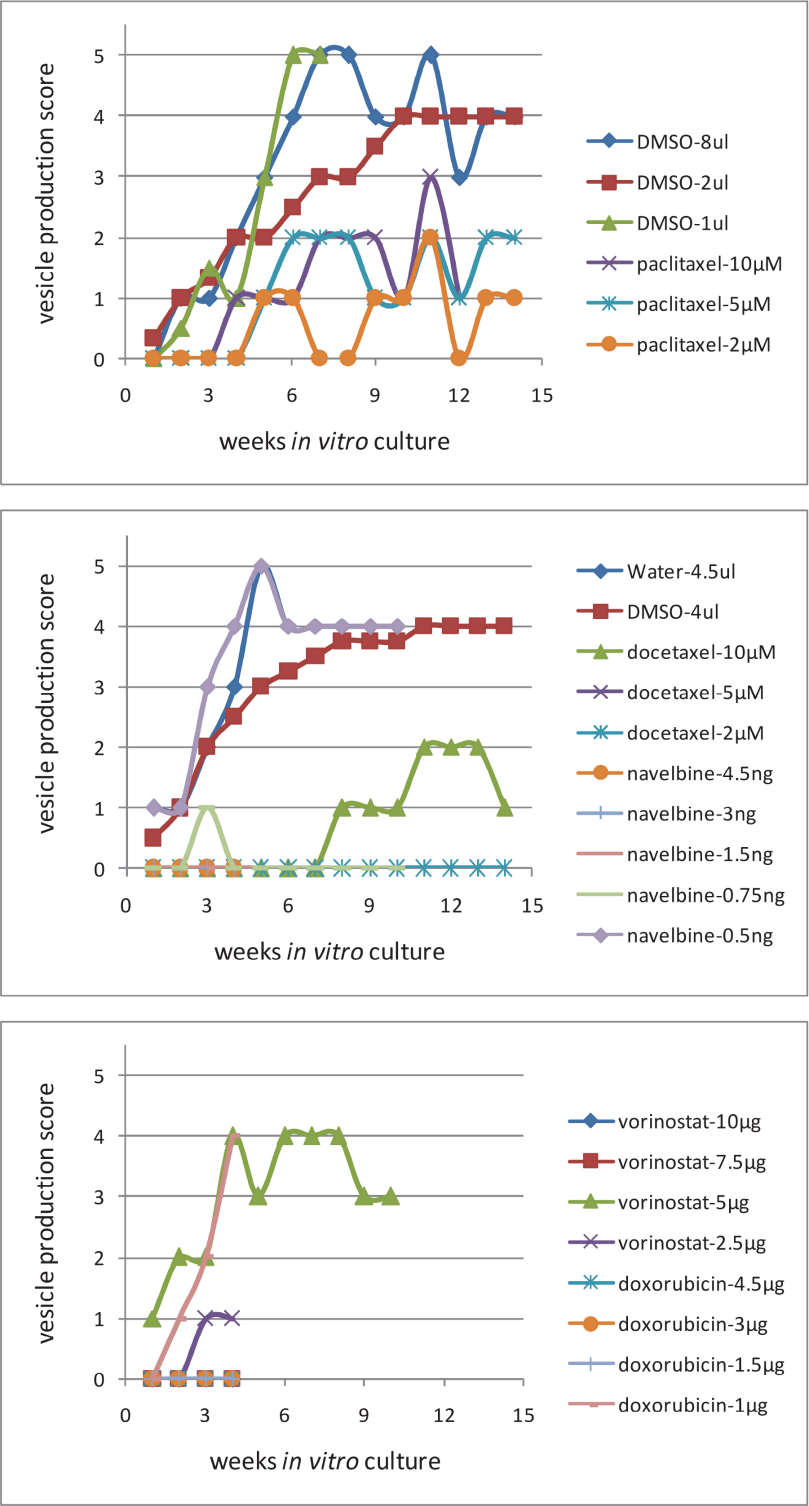
The *in vitro* production of vesicles from E. multilocularis metacestode tissues after *in vitro* drug-exposure *In vitro* cultured *E. multilocularis* metacestode tissue blocks (1 cm^3^) were exposed to 10 μM, 5 μM, 2 μM of docetaxel, to 4.5 μg/ml, 3 μg/ml and 1.5 μg/ml of doxorubicin, to 4.5 μg/ml, 3 μg/ml, 1.5 μg/ml and 0.75 μg/ml of navelbine, to 10 μM, 5 μM, and 2 μM, paclitaxel, to 10 μg/ml, 7.5 μg/ml, 2 μg/ml and 1 μg/ml of vorinostat (SAHA), and to DMSO (solvent control) at the indicated concentrations. The effects of these cytostatic compounds on the *in vitro* production by *E. multilocularis* vesicles was scored and studied for 14 weeks. The number of vesicles produced in culture was scored (Figure 5), i.e. Score 0 = no vesicle, Score 1 = very few vesicles (1–5), Score 2 = few vesicles (6–10), Score 3 = vesicles (11–20), Score 4 = vesicles (21–30), Score 5 = vesicles (>31).

*E. multilocularis* metacestode tissues were exposed to cytostatic drugs or drug-free culture media (control) for 1 week and maintained *in vitro* for another week drug-free, then the drug-exposed metacestode tissue culture media were changed weekly and the produced (“budded”) *E. multilocularis* vesicles (diameter 2 to 4 mm) collected, counted and vesicle production scored (Figure 4). Metacestodes exposed to docetaxel did not produce vesicles until seven weeks post exposure, thereafter, few vesicles (*n* = 1–5) were budding off the metacestode tissue blocks, and then vesicle production increased slightly (*n* = 6–10) from 10 weeks post exposure onwards. Already at 2–3 weeks post drug exposure, few vesicles (*n* = 1–5) were released from metacestode tissue blocks previously exposed to doxorubicin (4.5, 3 and 1.5 μg/ml), navelbine (4.5, 3, 1.5 and 0.75 μg/ml), paclitaxel (10 μM, 5 μM and 2 μM), vorinostat (SAHA) (10, 7.5, 2 and 1 μg/ml) (Figure 5), and the vesicle budding remained at this level until 14 weeks post drug exposure; thereafter cultures were ended. In Figure 5 the respective vesicle productions at the selected drug concentrations are shown. The *E. multilocularis* metacestode tissue cultures exposed to the DMSO solvent control budded off vesicles shortly after exposure, the release of vesicles continued to increase for weeks and reached at 7 weeks post DMSO exposure a plateau level of production (*n* = 20–30) which continued such until 14 weeks post drug exposure (Figure 5).

## DICUSSION

For AE patients treated with albendazole or mebendazole who experience severe side effects there are no alternative chemotherapeutics which reach beyond these classical benzimidazoles [1, 22]. We have applied gene microarray profiling of *E. multilocularis* metacestodes which showed strongly expressed human cancer-related genes suggesting similarities between metacestode proliferation and malignancies. In this pre-clinical study we exposed *in vitro E. multilocularis* metacestodes *in vitro* to cytostatic drugs, then implanted those drug-exposed parasite tissues into *Meriones unguiculatus* (gerbils), and studied *in vivo* parasite growth and proliferation by MRI and PET. *In vivo* growth and proliferation of *E. multilocularis* metacestode tissues was inhibited by docetaxel, while with paclitaxel and navelbine the *in vivo* growth of metacestodes was suppressed only until 3 months post infection, thereafter, parasite tissues enlarged in both drug-exposure groups. The histone deacetylase inhibitor vorinostat (SAHA), and doxorubicin which mediates DNA damage and inhibits DNA synthesis, were both not effective to inhibit *E. multilocularis* metacestode growth and proliferation, which is consistent with previous findings [4, 12].

New research approaches have been suggested which should explore new therapeutic molecules, exploit parasite gene signaling pathways, target *E. multilocularis* stem cells and dissect the metabolic metamorphosis of *E. multilocularis* metacestodes [23, 24]. There are several observations on antigenic similarities between *E. granulosus* and various tumor types [25, 26], and together with the finding on highly expressed tumor-related genes in metacestodes this suggested the evaluation of anti-cancer cytostatic drugs as treatment options for AE. The standard readout method for the assessment of drug efficacy is parasite weight determination subsequent to cyst resection from experimentally *E. multilocularis* infected mice, rats or gerbils. This method has pitfalls because necropsy can change the parasite mass due to cyst rupture and release of vesicle fluid, and the assessments of parasite viability based on parasite weights may not be exact because host connective tissue encapsulating the parasite makes a complete resection of the parasite mass difficult [21, 27]. Previous works on *in vitro* drug-exposure followed by *in vivo* growth monitoring, have exposed *E. multilocularis* metacestodes to mebendazole, then transferred these metacestodes into parasite-permissive gerbils *Meriones unguiculatus* where tissues did not grow [28], as determined by weight monitoring after dissection. Thus, the exposure of *E. multilocularis* metacestodes *in vitro* to mebendazole at concentrations above 0.1 μM was parasitocidal [28], and similarly effective was treatment using mefloquine (20 μM) against *in vitro* cultures of metacestodes but oral application of mefloquine to *E. multilocularis*-infected mice was ineffective, whereas oral albendazole application was highly effective [29]. Monitoring *in vivo* of the intra-peritoneal parasite growth without sacrificing the animal is possible by MRI and ultrasound allowing the assessments of parasite tissue volumes and its *in vivo* growth [11, 21], and by using PET tracers the metabolic activity of parasite tissues can be monitored, e.g. after application of therapeutic drugs [20]. The application of non-invasive imaging techniques, notably delayed [^18^F]FDG PET, greatly facilitated the differentiation between active and inactive liver lesions in AE patients, and the results suggested that the combination of delayed [^18^F]FDG PET and specific serology may help to prevent recurrences observed after premature interruption of treatment [19]. Further, imaging methods, using disease specific tracers for immuno-PET, have significant potential as effective tools to visualize infected tissues and cells, i.e. invasive pulmonary aspergillosis [18], and ligand-based targeting of specific cells or malignant tissues may help and guide surgeons to adequately resect infected while sparing critical tissues [30–32].

The taxanes paclitaxel, and docetaxel, a semi-synthetic analogue of paclitaxel, are proven anti-cancer drugs and FDA-approved formulations of first line against advanced prostate cancer. Paclitaxel and docetaxel have a similar mechanism of action, they promote tubulin assembly and inhibit microtubule disassembly, stabilizing microtubule polymerization and thus blocking cells in the G2/M phase of the cell cycle thus triggering the signaling pathway that leads to apoptosis. Docetaxel is effective against tumor cells by inducing cell death, it inhibits the transcription of androgen receptors thus improving survival in metastatic hormone-resistant prostate cancer and also with tumors at earlier stages [33]. Navelbine is approved for the treatment of non-small cell lung cancer and metastatic breast cancer [34, 35]. Paclitaxel and docetaxel distribute into most tissues of mice and rats, including tumor tissue, but despite similarity in chemical structures their metabolic profile is distinct. Whereas paclitaxel metabolism is largely species dependent, docetaxel metabolism is similar across species, and for both taxanes, hepatobiliary excretion is the major pathway of elimination, and a major fraction of the dose is excreted in feces as parent drug or hydroxylated metabolites [36]. Albendazole remains the most common and effective treatment for AE, it targets tubulin but has its limitation, such as poor solubility and intestinal absorption and often there is no complete recovery after treatment. High dosage and lifelong uptake is required for albendazole in AE patients, which may lead to severe adverse effects [22]. Thus, less uptake time and high efficiency with paclitaxel and docetaxel may decreases the adverse effects and lead to potential treatments.

Recently, the anti-cancer drug bortezomib, a proteasome inhibitor developed for the chemotherapy of myeloma, displayed high anti-metacestodal activity, and Balb/c mice experimentally infected with *E. multilocularis* metacestodes presented with reduced parasite weights, but bortezomib treatment induced adverse effects such as diarrhea and neurological symptoms [12]. Previously, we found that navelbine suppressed *in vivo E. multilocularis* metacestode growth and proliferation [11], and the present results show that docetaxel visibly, and paclitaxel to a lesser extent, inhibited parasite growth. Similar results were reported by Pensel PE *et al.*, who showed the paclitaxel can inhibit the survival of larval cell, protoscoleces and metacetodes of *Echinococcus granulosus* [9]. There are clinically relevant differences between docetaxel and paclitaxel, docetaxel is more cytotoxic than paclitaxel against a variety of murine and human tumor cell lines [37]. Both have been serving as important drugs for the treatment of various cancers, but drug resistance imposes limitations in their application since both have high affinity for multidrug-resistance proteins, in particular the drug efflux pump P-glycoprotein [38].

In conclusion, our observations advocate for drug combinations to be applied in experimental pre-clinical studies; which may provide essential information on their efficacy against *E. multilocularis* metacestodes, and ultimately this may translate into new treatment options against alveolar echinococcosis.

## MATERIAL AND METHODS

### Animal model of alveolar echinococcosis

All experiments were performed according to the German Animal Protection Law with permission from the Regierungspräsidium Tübingen as per guidelines from the European Health Law of the Federation of Laboratory Animal Science Associations (FELASA). Ten-week-old female gerbils (*Meriones unguiculatus*) were purchased from Charles River Laboratories (Sulzfeld, Germany) or bred in our animal facility. The animals were kept under standardized and sterile environmental conditions (20° C ± 1° C room temperature, 50% ± 10 % relative humidity, 12 h light-dark cycle) and received food and water *ad lib*.

*E. multilocularis* metacestode tissue was routinely maintained in gerbils using a modified method of serial implantation of parasite tissue as described previously [39, 11]. The metacestode tissue was passed through a metal sieve with 1 mm^2^ width. *M. unguiculatus* (gerbils) were anesthetized with 2% isoflurane mixed with 100% oxygen and 0.5ml of the metacestode tissue cell suspension was injected into the peritoneum of each gerbil. After sufficient growth of the metacestode tissue, the gerbils were euthanized with CO_2_, and the parasite tissues were removed and used for *in vitro* culture assays and for *in vivo* transfer and maintenance of the *E. multilocularis* metacestodes.

### *In vitro* culture of E. multilocularis metacestodes

For *in vitro* cultivation of *E. multilocularis*, metacestode blocks were freshly and aseptically removed from the peritoneal cavity of experimentally infected gerbils (*Meriones unguiculatus*) [11, 39] and incubated with RPMI 1640 medium supplemented with 10% FCS and 1% penicillin/streptomycin (Biochrom GmbH, Berlin, Germany) at 37° C and 5% CO_2_. Medium was changed once a week for all cultures.

### Purification of *E. multilocularis* metacestodes total RNA, cDNA generation, labeling and oligonucleotide hybridization and microarray

*E. multilocularis* metacestodes were cultured *in vitro* as described [11]. Metacestode tissue blocks which were not drug-exposed were snap frozen in liquid nitrogen, the deep frozen tissues were minced and homogenized with a tissue grinder, total RNA was purified by RNeasy Mini Kit (Qiagen, Hilden, Germany). The RNA was quantified with a Nanodrop UV spectrofluorometer and quality of RNA determined by Agilent Bioanalyzer 2100 (Agilent, CA, USA). Double-stranded cDNA was synthesized from 100 ng of total RNA and subsequently linearly amplified and biotinylated using the GeneChip^®^ WT cDNA Synthesis and Amplification Kit (Affymetrix, Santa Clara, CA, USA) according to the manufacturer’s instructions. Microarrays were analyzed with 15 μg of labeled and fragmented cDNA hybridized to GeneChip^®^ HumanGene 1.0 ST arrays (Affymetrix). After hybridization, the arrays were washed and stained in a Fluidics Station 450 (Affymetrix) with the recommended washing procedure. Biotinylated cDNA bound to target molecules was detected with streptavidin-coupled phycoerythrin, biotinylated anti-streptavidin IgG antibodies and again streptavidin-coupled phycoerythrin according to the protocol. Arrays were scanned using the GCS3000 GeneChip scanner (Affymetrix) and AGCC 3.0 software. Scanned images were subjected to visual inspection to check for hybridization artifacts and proper grid alignment and analyzed with Expression Console 1.0 (Affymetrix) to generate report files for quality control. Normalization of raw data was performed by the Partek Software 6.6, applying an RMA (Robust Multichip Average) algorithm. For analysis, microarray hybridization data were converted to signal values using ArrayAssist 3.4 (Stratagene), and the signal strength of hybridization of the *E. multilocularis* cDNA samples to the human micro-array chip greater than 100 above background were selected. Two *E. multilocularis* metacestode samples, which were cultured as described above without having been exposed to anti-cancer drugs, were applied and the hybridization signals of both samples and their mean signal strength were aligned and are shown in Supplementary Table 1.

### Selection of cytostatic drugs

Gene expression profiling in *E. multilocularis* metacestodes showed strongly expressed human cancer-related genes which suggested similarities between *E. multilocularis* metacestode proliferation and cancer progression and tissue metastases. The signal strength of hybridization to human genes was prominent for member of the RAS oncogene family, the folate receptor, the eukaryotic translation elongation factor 1 alpha 1, tubulin, aquaporin, calreticulin, and synuclein alpha (Supplementary Table 1). Based on these hybridization signals and specific gene expression FDA-approved formulations against advanced cancer were selected for the *in vitro* exposure of *E. multilocularis* metacestodes. Taxanes were selected for their capacity to stabilize microtubule polymerization thus blocking the cell cycle which leads to apoptosis. vorinostat (SAHA) was selected for inducing cell cycle arrest, doxorubicin for its capacity to mediate DNA damage and to inhibit DNA synthesis and proliferation, and both SAHA and doxorubicin may act in synergy to inhibit growth of tumor cells.

### Exposure of E. multilocularis to cytostatic drugs

*In vitro* cultured *E. multilocularis* metacestode tissue blocks (1 cm^3^) were exposed to docetaxel, paclitaxel, navelbine, doxorubicin, or vorinostat in concentrations according to their recommended dosage for cancer treatment in humans. Metacestode tissues were exposed *in vitro* to 10 μM, 5 μM, 2 μM of docetaxel (Sigma-Aldrich; #01885-F), to 4.5 μg/ml, 3 μg/ml and 1.5 μg/ml of doxorubicin (Sigma-Aldrich; #44583), to 4.5 μg/ml, 3 μg/ml, 1.5 μg/ml and 0.75 μg/ml of navelbine (Pierre Fabre, Freiburg, Germany, 10 μg/ml; UKT#2698), to 10 μM, 5 μM and 2 μM paclitaxel (Sigma-Aldrich; #T7191), to 10 μg/ml, 7.5 μg/ml, 2 μg/ml and 1 μg/ml of vorinostat (SAHA) (Sigma-Aldrich; SML0061) and to DMSO (Sigma-Aldrich; #D8418) (0.1%, 0.05%; solvent control) at the indicated concentrations, and the effects of these cytostatic compounds on growth and proliferation of *E. multilocularis* metacestodes were evaluated *in vitro* and *in vivo*.

### E. multilocularis infection *in vivo*

Metacestodes of *E. multilocularis* were cultured *in vitro* by established techniques [11] and the infection of *M. unguiculatus* was carried out according to the previous study [11]. In brief, metacestodes were exposed to cytostatic drugs or drug-free culture media (as above) for 1 week and then *in vitro* culture continued for another week in drug-free culture media. Thereafter, metacestode tissue blocks were split in half, one for further *in vitro* culture and monitoring of vesicle production and the other half was used to prepare the metacestode suspension for intra peritoneal injection (i.p.) in *M. unguiculatus*. The growth of drug-treated and untreated *E. multilocularis* metacestodes was monitored *in vivo*. All animals were examined for metacestode growth by *in vivo* magnetic resonance imaging (MRI) and tracer-guided positron emission tomography (PET). After the last PET scan at 5 months p.i., *M. unguiculatus* were autopsied, the Em-metacestode tissues removed and weighted. Most metacestode tissues were recovered from the peritoneal cavity either as singularly isolated masses or dissected from liver, kidney or gut tissues.

### Drug-exposure of *in vitro* cultured E. multilocularis metacestodes

Drug-exposed metacestode tissue blocks were cultured *in vitro* as described previously [11]. One half of the drug exposed parasite tissue was incubated with RPMI 1640 medium supplemented with 10% FCS and 1% penicillin/streptomycin (Biochrom GmbH, Berlin, Germany) at 37° C and 5% CO_2_. The culture medium was changed once a week and *E. multilocularis* vesicles were collected from the cell culture and the number of produced vesicles (diameter 2 to 4 μM) counted. The number of vesicles produced in culture was scored (Figure 5), i.e. Score 0 = no vesicle, Score 1 = very few vesicles (1–5), Score 2 = few vesicles (6–10), Score 3 = vesicles (11–20), Score 4 = vesicles (21–30), Score 5 = vesicles (>31).

### *In vivo* volumetric quantization of parasite growth

For the volumetric evaluation of *E. multilocularis* metacestode growth in infected *Meriones*, *in vivo* MRI was performed using a 7T, 300 Mhz small animal MR tomograph (Bruker Biospin MRI GmbH, Ettlingen, Germany) for the acquisition of anatomical information. The animals were anesthetized during the measurements with 1.5% isoflurane mixed with 100% oxygen under respiration monitoring. The images were acquired using a T2 fat saturated 3D sequence with a TE/TR of 90.51/1800.000 ms and data were analyzed using Inveon Research Workplace software (IRW, Siemens Preclinical Solutions, Knoxville, TN, USA). Parasite tissues were delineated from other organs based on the anatomical information obtained from the MR images and marked as regions of interest (ROIs) and quantified volumes are expressed as cubic millimeters.

### PET tracer production

Fluorine-18 was produced as ^18^F-fluoride at the PET trace cyclotron (General Electric Medical Systems, GEMS, Uppsala, Sweden) using the ^18^O(p,n)^18^F nuclear reaction, and [^18^F]FDG was synthesized as described [40].

### PET/MR imaging

Simultaneous PET/MR imaging was performed with *E. multilocularis* infected gerbils 5 months p.i. *In vivo* bio-distribution of the PET tracer [^18^F]FDG was assessed using a small animal PET insert (Bruker Biospin GmbH, Ettlingen, Germany) yielding a spatial resolution of approximately 1.3 mm in the reconstructed images [41]. All animals were shortly anaesthetized with isoflurane and *i.v.* injected with 10–12 MBq of the tracer via a lateral tail vein. Static (10 min) PET scans were acquired after the injection of the tracer. During PET/MR imaging, the animals were anesthetized with 1.5% isoflurane mixed with 100% oxygen. Anesthesia was monitored by measuring the respiratory frequency, and the body temperature was kept at 37°C using a heating pad. PET data were acquired in list-mode, histograms collected in one 10 min time frame and reconstructed using an iterative ordered subset expectation maximization (OSEM) algorithm. No attenuation correction was applied. MR imaging was performed as described above on a 7T, 300 Mhz dedicated small animal MR tomograph obtaining anatomical information for parasite delineation. In addition to the T2 fat saturated 3D sequence, a T1 3D fast low angle shot (FLASH) sequence with a TE/TR of 6.000/30.000ms was performed. PET images were normalized to each other, subsequently fused to the respective MR images and analyzed using IRW. ROIs were drawn around the respective tissue based on the anatomical information obtained from the MR images. Absolute quantification of the PET data is expressed as percentage of the injected dose per cubic centimeter (%ID/cc). After the final PET scan, all animals were euthanized under deep anesthesia and parasite tissue was removed and weighted.

### Statistical analysis

For the analysis of microarray data, significance was calculated using a *t*-test without corrections for multiple testing selecting all transcripts with a minimum change in expression level of 1.5-fold together with a *p*-value of less than 0.05. The signal strength of hybridization of the *E. multilocularis* cDNA samples to the human microarray chip greater than 100 above background were selected.

## SUPPLEMENTARY MATERIALS TABLE



## Abbreviations

AE: Alveolar echinococcosis
BMZ: benzimidazole
Em: *Echinococcus multilocularis*
FDA: Food and Drug Administration
i.p.: intra peritoneal
MRI: Magnetic resonance imaging
MIP: maximum intensity projections
n.d.: non detected
NMR: nuclear magnetic resonance
PET: positron emission tomography
p.i.: post infection
[^18^F]FDG: 2-deoxy-2-[^18^F]-fluoro-D-glucose
%ID/cc: percentage of the injected dose per cubic centimeter
