# Supplementary material for: The effects of taxanes, vorinostat and doxorubicin on growth and proliferation of Echinococcus multilocularis metacestodes assessed with magnetic resonance imaging and simultaneous positron emission tomography
Source: Oncotarget. 2018 Jan 10;9(10):9073–87. doi: 10.18632/oncotarget.24142 (PMC5823665; doi:10.18632/oncotarget.24142)
Supplement: Supplementary file 1 [file oncotarget-09-9073-s001.pdf]

The effects of taxanes, vorinostat and doxorubicin on growth and proliferation of *Echinococcus multilocularis* metacestodes assessed with magnetic resonance imaging and simultaneous positron emission tomography

SUPPLEMENTARY MATERIALS

Supplementary Table 1: The signal strength of hybridization of *Echinococcus multilocularis* cDNA to human microarray chip

Genbank Accession No.	Signal Strength Sample 1	Signal Strength Sample 2	Mean Signal Strength (S1 + S2)	Gene Title
BC002778	96,3	102	99	myosin light chain 2, precursor lymphocyte-specific
U81961	104,1	88,4	96	sodium channel, nonvoltage-gated 1 alpha
W94546	87,5	103,5	96	hypothetical protein 284297
AA398062	100,9	87,03	94	aminopeptidase-like 1
AI251399	71,3	114,8	93	protein kinase D2
NM_000748	115,2	70,3	93	cholinergic receptor, nicotinic, beta polypeptide 2 (neuronal)
AK096064	155,9	29,2	93	---
AI304355	101,7	80,81	91	Chromosome 1 open reading frame 78
AW248552	143,2	32,9	88	NOL1/NOP2/Sun domain family, member 5
M80469	102,7	72,89	88	HLA-G histocompatibility antigen, class I, G /// major histocompatibility complex, class I, H (pseudogene)
AK024602	128,3	38,5	83	CDNA: FLJ20949 fis, clone ADSE01902
NM_002587	100	64,3	82	protocadherin 1 (cadherin-like 1)
BG701300	106,6	53,5	80	hypothetical gene supported by BC030123
NM_002375	109,1	40,8	75	microtubule-associated protein 4
NM_024671	104,5	42,3	73	hypothetical protein FLJ23436
AL390137	104	42	73	Eukaryotic translation initiation factor 3, subunit 10 theta, 150/170kDa
NM_032887	112,6	26,6	70	hypothetical protein MGC16037
BC014556	100,1	36,7	68	hypothetical protein FLJ35390
X07618	104,7	30,1	67	---
AA362254	119,8	13,8	67	CDNA FLJ30424 fis, clone BRACE2008881, weakly similar to ZINC FINGER PROTEIN 195
NM_022830	21	109,2	65	RNA binding motif protein 21
NM_006316	110,1	19,4	65	v-myc myelocytomatosis viral related oncogene, neuroblastoma derived (avian) opposite strand

The signal strength of hybridization of metacestode cDNA (Sample 1 and 2) to oligonucleotides on human microarray, the mean hybridization values (S1 + S2), the Genbank accession number and the Gene Title are shown. From *E. multilocularis* metacestodes, which were never drug-exposed, total RNA was purified and double-stranded cDNA synthesized, linearly amplified, biotinylated and the fragmented cDNA hybridized to GeneChip® HumanGene 1.0 ST microarrays (Affymetrix). Biotinylated cDNA bound to target molecules on microarrays was detected with streptavidin-coupled phycoerythrin, biotinylated anti-streptavidin IgG and streptavidin-coupled phycoerythrin using the GCS3000 GeneChip scanner (Affymetrix) and AGCC 3.0 software. Scanned images were analyzed with Expression Console 1.0 (Affymetrix) by applying an RMA (Robust Multichip Average) algorithm. Microarray hybridization data were converted to signal values using ArrayAssist 3.4 (Stratagene), and the signal strength of hybridization of the *E. multilocularis* cDNA samples to the human micro-array chip greater than 100 above background were selected. Two *E. multilocularis* metacestode samples (Sample 1 and Sample 2) were applied to human microarray analysis, and hybridization signals of either sample greater than 100 and their mean signal strength were aligned and are shown.
